# The appearance of newly identified intraocular lesions in Gaucher disease type 3 despite long-term glucocerebrosidase replacement therapy

**DOI:** 10.3109/03009734.2016.1158756

**Published:** 2016-04-06

**Authors:** Nadia Sawicka-Gutaj, Maciej Machaczka, Izabela Kulińska-Niedziela, Jadwiga Bernardczyk-Meller, Paweł Gutaj, Jerzy Sowiński, Marek Ruchała

**Affiliations:** a Department of Endocrinology, Metabolism and Internal Medicine, Poznan University of Medical Sciences, Poznan, Poland;; b Hematology Center Karolinska and Department of Medicine at Huddinge, Karolinska Institutet, Karolinska University Hospital Huddinge, Stockholm, Sweden;; c Heliodor Swiecicki Clinical Hospital, Ophthalmology Outpatient Clinic, Poznan University of Medical Sciences, Poznan, Poland;; d Department of Ophthalmology, NZOZ ‘OCU SERVICE’, Poznan, Poland;; e Department of Obstetrics and Women’s Diseases, Poznan University of Medical Sciences, Poznan, Poland

**Keywords:** Enzyme replacement therapy, Gaucher disease type 3, intraocular lesions, neuronopathic, optical coherence tomography, retina

## Abstract

**Background:**

Gaucher disease (GD) is an autosomal recessive lipid storage disorder caused by the deficient activity of the lysosomal enzyme glucocerebrosidase. The presence of central nervous system disease is a hallmark of the neuronopathic forms of GD (types 2 and 3). Intraocular lesions (e.g. corneal clouding, retinal lesions, and vitreous opacities) have been infrequently reported in GD type 3 (GD3). Moreover, there are virtually no published data on the occurrence and natural course of intraocular lesions in GD3 patients treated with enzyme replacement therapy (ERT).

**Case presentation:**

We describe the case of a 26-year-old Polish male with L444P homozygous GD3 (mutation c.1448T > C in the *GBA1* gene) who developed fundus lesions despite 10 years of ERT. At the age of 23 years, a spectral domain optical coherence tomography (OCT) examination was performed which disclosed the presence of discrete lesions located preretinally, intraretinally in the nerve fiber layer, and in the vitreous body. A 3-year follow-up OCT examination has not shown any significant progression of the fundus lesions.

**Conclusions:**

To the best of our knowledge, this is the first published report describing the occurrence of newly identified retinal and preretinal lesions occurring during long-term ERT in GD3. We recommend that a careful ophthalmic assessment, including a dilated fundus examination, should be included as part of annual follow-up in patients with GD3. Further studies are needed to understand the nature and clinical course of these changes and whether or not these intraocular findings have any predictive value in the context of neurologic and skeletal progression in GD3.

## Introduction

Gaucher disease (GD) is an autosomal recessive lipid storage disorder caused by the deficient activity of the lysosomal enzyme glucocerebrosidase (1). There are more than 300 known mutations in the *GBA1* gene (1q21) that can cause GD, of which the c.1226A > G (N370S) and the c.1448T > C (L444P) mutations are the most prevalent (2). Decreased glucocerebrosidase activity results in cytomorphologically noticeable lysosomal accumulation of glucosylceramide in cells of the monocyte-macrophage system such as the spleen, liver, and bone marrow (3,4).

GD is known for its phenotypic diversity, and the clinical picture can vary from severe, lethal cases diagnosed before or shortly after birth to cases where patients are completely asymptomatic (5–11). Three clinical types of GD are distinguished based on the absence (type 1) or presence (types 2 and 3) of neurologic symptoms and the dynamics of developing clinical signs (5–7). Globally, the most prevalent form of GD is non-neuronopathic GD type 1 (GD1) with the main symptoms of thrombocytopenia, anemia, hepatosplenomegaly, and bone manifestations (6,8–11). In addition to the symptoms mentioned for GD1, the presence of central nervous system disease is a hallmark of the neuronopathic forms of GD (5,7,12).

Horizontal supranuclear gaze palsy is one of the earliest signs of the neuronopathic forms of GD (5,7,13,14). Other typical ocular manifestations in neuronopathic GD include oculomotor apraxia and convergent squint (7,13,14). Intraocular manifestations such as corneal clouding, retinal lesions, and vitreous opacities have been reported in GD type 3 (GD3), but they have not been characterized in detail (15–17). Moreover, there are virtually no published data regarding the occurrence and natural course of intraocular lesions in patients with GD3 treated with enzyme replacement therapy (ERT).

Here, we present the case of a young patient with GD3 who developed intraocular lesions despite 10 years of glucocerebrosidase replacement. In addition, we also provide results of his 3-year follow-up examination using spectral domain optical coherence tomography (OCT).

## Case report

A 26-year-old Polish male was diagnosed with GD at the age of 3 years. His past medical history was significant for splenomegaly, identified at the age of 12 months, and severe pancytopenia. Gaucher cells were not found in aspiration biopsies of the bone marrow. He was splenectomized at the age of 3 years due to massive splenomegaly, and the diagnosis of GD was established by the presence of low activity of glucocerebrosidase in peripheral blood leukocytes. Further direct DNA sequencing revealed the homozygous mutation c.1448T > C in the *GBA1* gene (i.e. mutated alleles L444P/L444P), which suggested GD3. At nearly 6 years of age, he developed avascular necrosis of the left femoral head.

**Figure 1.**
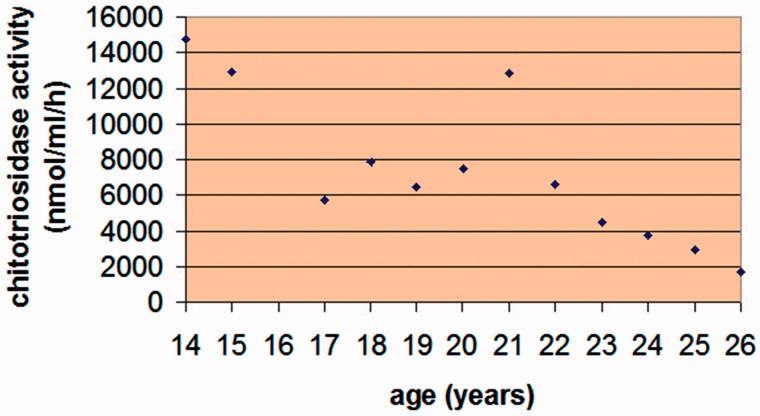
The patient’s plasma chitotriosidase activity measured annually since the age of 14 years. The patient’s ERT was discontinued for 4 months at 20 years of age, causing a sharp increase in his plasma chitotriosidase activity.

The patient started intravenous ERT with macrophage-targeted recombinant glucocerebrosidase at the age of 8 years, immediately after ERT became available in Poland. At the age of 20, his ERT was discontinued for 4 months during the unintentional world-wide imiglucerase (Cerezyme®, Genzyme Corporation, Cambridge, MA, USA) supply shortage (12). This was followed by a rapid increase in plasma chitotriosidase activity (Figure 1), pathological bilateral forearm fractures and fractures in the left brachial bone and both fibulae. The fractures healed slowly, caused chronic pain, and created difficulties in walking for the patient. Currently, the patient is receiving infusions of imiglucerase at a dose of 56 units/kg of body weight, administered every other week.

### Ocular findings

The patient had annual ophthalmologic follow-up examinations from diagnosis. His eye fundus examinations were normal until lesions were first noted at the age of 18 (i.e. 15 years from his diagnosis of GD and 10 years from the start of ERT). The fundus lesions were described as white spots located peripherally and in the posterior pole.

**Figure 2.**
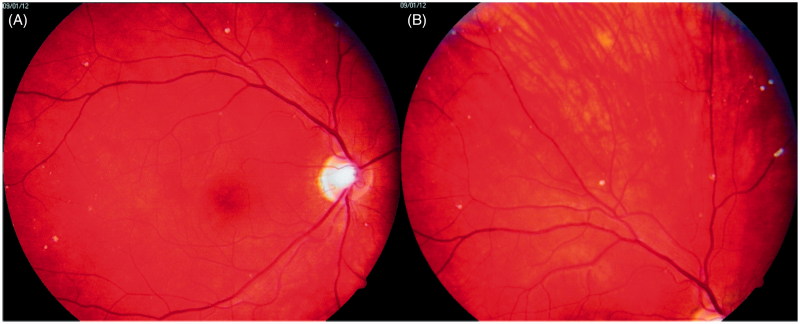
Photographs of the patient’s right eye fundus. A: posterior pole; B: periphery of the fundus. White spots overlie retinal vessels.

**Figure 3.**
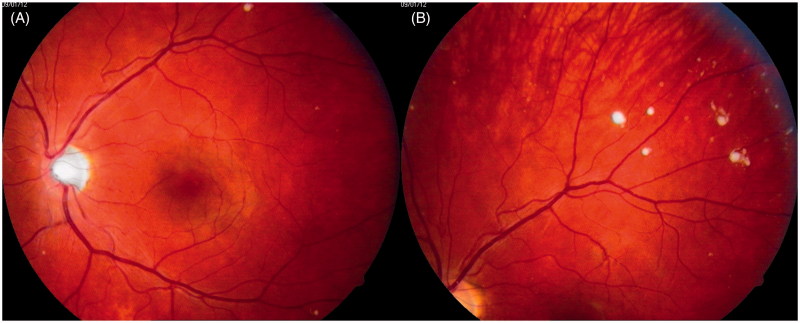
Photographs of the patient’s left eye fundus. A: posterior pole; B: periphery of the fundus. White spots present along retinal vessels with a distribution of changes similar to that seen in the right eye.

**Figure 4.**
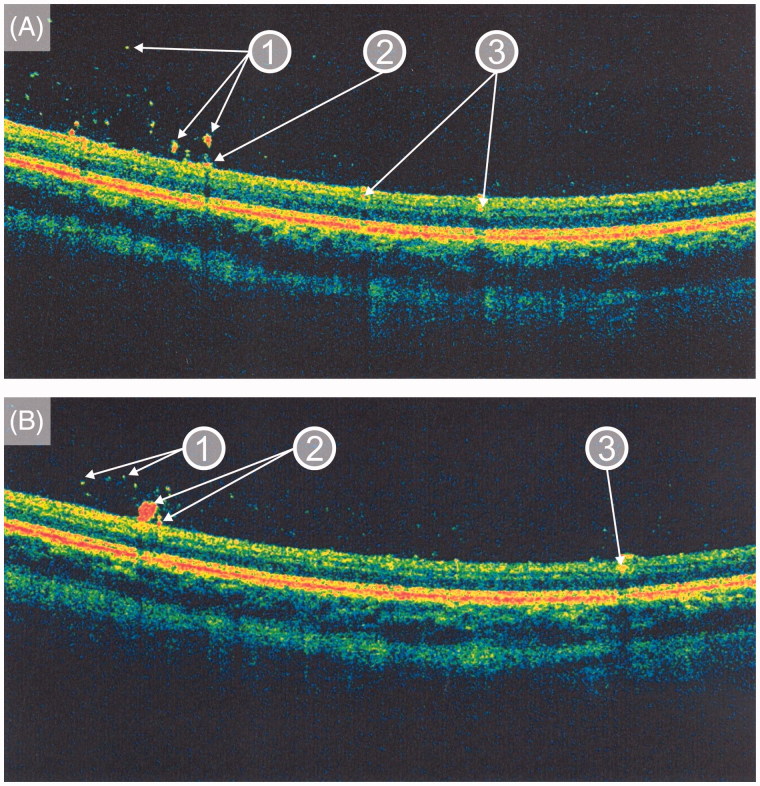
Optical coherence tomography images taken in January 2012 (A, B) showing (1) intravitreal, (2) preretinal, and (3) intraretinal (in the nerve fiber layer) locations of the deposits in the presented patient with Gaucher disease type 3.

In January 2012, when the patient was 23 years old, his eye changes were documented in photographs (Figures 2 and 3). At that time, the lesions were located on the surface of the retina and in the preretinal part of the vitreous body. OCT confirmed the presence of discrete lesions located preretinally, intraretinally in the nerve fiber layer, and in the vitreous body (Figure 4). Electroretinography (ERG) did not show any pathological pattern, thereby indicating normal retinal function.

A recent ophthalmologic examination (performed in December 2014) showed a best-corrected visual acuity of 20/20 bilaterally and an intraocular pressure of 19 mmHg (normal values: 11–21 mmHg). Ocular motility examination demonstrated limitations in abduction of both eyes. Dilated fundoscopy revealed optic discs with well-defined borders and pink color in both eyes. The maculae were normal. Further examinations showed yellowish-white punctuate deposits in the posterior pole and the periphery of the fundus bilaterally. A 3-year follow-up OCT examination was performed in January 2015, and no signs of progression of the fundus lesions were evident.

## Discussion

GD affects less than 1/50,000 population but can be found in all ethnic groups around the world. In Poland, the overall prevalence of GD is not yet established; however, in Sweden the prevalence is approximately 1/170,000 individuals (6), slightly lower than that reported in other Western countries but 2.5 times higher than in other Nordic countries (1,6).

Although the neuronopathic forms are the rarer variants of GD (less than 5% of all GD cases), an endemic cohort of Swedish patients with chronic neuronopathic GD3 lives in the county of Norrbotten in Northern Sweden (12). It is worth noting that, in both Sweden and Poland, GD3 comprises approximately 40% of all known cases of GD (12,14).

The incidence of vitreous opacities was found to be 3% in a series of 80 patients with GD1 (18). It was postulated that only those GD1 patients who underwent splenectomy have a tendency to form vitreous aggregates. Similarly, it was suggested that preretinal and retinal white spots, which occur mostly in splenectomized patients with GD, represent an unusual systemic location of lipid-laden macrophages, so-called ‘Gaucher cells’, due to the higher levels of circulating glucosylceramide (15,19,20). Such results found on histopathologic examination of the fundus lesions in GD have only been published in Japanese (an autopsy study) and only mentioned in the English medical literature (15).

In 2004, Shrier and colleagues reported a case of vitrectomy in a 20-year-old white woman with GD (20). The patient’s GD type and *GBA1* mutations were not reported. Her vitreous aspirate specimen disclosed some degenerated and a few typical Gaucher cells. The entire vitrectomy specimen, analyzed after centrifugation, showed a large amount of glucosylceramide.

Intraocular lesions have been infrequently reported in patients with GD3 (16,21). In the case presented here, the reported lesions appeared despite long-term glucocerebrosidase replacement therapy. One possible explanation for this finding is the large molecular size of recombinant glucocerebrosidase, which prevents it from crossing the blood–brain barrier, therefore not allowing it to reach the eye, as postulated by Coussa and colleagues (21). Similar to the patient with GD3 described by Coussa et al., our patient had normal visual acuity and ERG findings. However, Seidova et al. reported preretinal lesions and subtle retinal dysfunction on ERG in a splenectomized patient with GD1 (19).

There is only one published report, by Sheck et al., describing preretinal opacities seen with OCT in a 14-year-old girl with GD3 treated with ERT (16). OCT is a non-contact optical device that provides cross-sectional images and quantitative analysis of the ocular tissues (22). Unfortunately, Sheck et al. did not provide any information as to whether these preretinal changes were present before the start of ERT, and they have not reported any OCT follow-up data (16). Based on the OCT findings, they proposed that intraocular opacities in GD are preretinal, located at the vitreo-retinal interface associated with localized posterior vitreous detachments rather than vitreous opacities as previously suggested (16). In contrast to Sheck and colleagues, we have shown with OCT the presence of lesions on the surface of the retina and in the vitreous body.

Recently, McNeill and colleagues published the results of a pilot study in which they suggest that thinning of the retinal ganglion cell layer may be associated with early neurodegeneration in patients with GD1 (23). We speculate whether retinal and preretinal deposits could be an early warning sign of disease progression in GD. It is worth noting that the appearance of the intraocular lesions seen in our patient preceded his severe skeletal complications by 2 years, which supports this hypothesis. One might argue that interruption of imiglucerase therapy alone could cause the aforementioned bone fractures. Although this may be true with respect to a long-lasting interruption of ERT, it is doubtful whether missing only eight doses of imiglucerase would result in such severe bone complications so quickly in the absence of obvious skeletal disease progression in this patient.

Thus, we recommend that a careful ophthalmic assessment including a dilated fundus examination be included as part of annual follow-up in patients with GD3. When intraocular lesions are detectable in GD3, a subsequent OCT examination would provide further information on the exact location of the deposits in relation to retinal architecture.

To the best of our knowledge, this is the first published report on the occurrence of newly identified retinal and preretinal lesions occurring during long-term ERT in GD3. Our case report highlights the fact that OCT is helpful in the follow-up of intraocular lesions detected in patients with GD3. Further studies are needed to explain better the nature and clinical course of these changes. It would be especially valuable to know whether or not these intraocular findings have any predictive value in the context of neurologic and skeletal progression in GD3.
